# Chelation Drives Surface Substitution in Hybrid‐MXenes

**DOI:** 10.1002/anie.6432447

**Published:** 2026-07-20

**Authors:** Vikash Khokhar, Young‐Hwan Kim, Abu Rashed Md. Shawon, Fatemeh Karimi, Danial Zangeneh, Yongqiang Cheng, Chang Liu, Murillo Longo Martins, Alexander S. Filatov, Robert F. Klie, Aaron J. Rossini, John S. Anderson, Dmitri V. Talapin, De‐en Jiang

**Affiliations:** ^1^ Interdisciplinary Materials Science Vanderbilt University Nashville Tennessee USA; ^2^ Department of Chemistry Pritzker School of Molecular Engineering and James Franck Institute University of Chicago Chicago Illinois USA; ^3^ Division of Materials Science and Engineering Ames National Laboratory Ames Iowa USA; ^4^ Department of Chemistry Iowa State University Ames Iowa USA; ^5^ Department of Physics University of Illinois Chicago Chicago Illinois USA; ^6^ Neutron Scattering Division Oak Ridge National Laboratory Oak Ridge Tennessee USA; ^7^ Chemical Science Division Oak Ridge National Laboratory Oak Ridge Tennessee USA; ^8^ Department of Chemical and Biomolecular Engineering Vanderbilt University Nashville Tennessee USA

**Keywords:** amine ligands, chelation, density functional theory, denticity, deprotonation, inelastic neutron scattering, mxenes, surface modification

## Abstract

Hybrid organic–inorganic MXenes (*h*‐MXenes) offer a versatile platform for tailoring the surface chemistry of two‐dimensional transition‐metal carbides and nitrides through covalently bound organic ligands. Although monodentate amido/imido functionalization has been demonstrated previously, chelating ligand binding has remained unexplored. Here, we report the synthesis of Ti_3_C_2_(en)_x_ *h*‐MXenes via substitution of Br terminations in Ti_3_C_2_Br_2_ with deprotonated ethylenediamine (en). By varying the amount of n‐BuLi used for en deprotonation, the surface coordination evolves from predominantly monodentate to bidentate binding. This transition is evidenced by a contraction of the interlayer spacing and attenuation of the ─NH_2_ signal in x‐ray photoelectron spectroscopy. Solid‐state NMR reveals that bidentate coordination dominates when 4 equiv. of n‐BuLi is employed, while inelastic neutron scattering, supported by simulated vibrational spectra, provides independent confirmation of the bidentate binding motif. Density functional theory calculations show that bidentate en is significantly more stable than monodentate configurations for replacing Br terminations. Ab initio molecular dynamics further reveal dynamic surface chemistry involving proton transfer, β‐H elimination, surface imine–Ti bond formation, and partial reversion to monodentate coordination. These findings establish ligand denticity as a new design parameter for engineering MXene surface chemistry and tuning material properties.

## Graphical abstract

We demonstrate ethylenediamine‐derived bidentate binding on Ti_3_C_2_ by controlled deprotonation of ─NH_2_ groups during reaction with Ti_3_C_2_Br_2_. XRD, XPS, ssNMR, STEM, DFT, and INS confirm the bidentate coordination and the superior stability of chelating modes, while AIMD reveals proton transfer and new surface imine–Ti bonding mode.

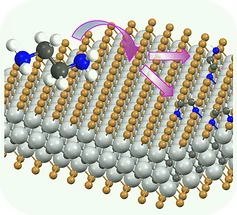

## 1 Introduction

MXenes are 2D transition metal carbides/nitrides [1] that are used in a wide range of applications, including energy storage and electrochemistry [2, 3, 4, 5], catalysis [6, 7, 8, 9, 10], optoelectronics [11, 12], sensors and actuators [13, 14, 15], lubricants [16, 17], wireless communications and antennas [18], electromagnetic interference shielding [19, 20, 21, 22], and biomedical applications [23, 24, 25]. Terminating groups on MXene surfaces were initially limited to O, OH, and F from traditional synthesis using HF etching of MAX phases, but have since been extended to pure halides using molten salt etching [26] and direct MXene syntheses [27]. The resulting halide terminations, like Cl or Br, are synthetically enabling as they are labile enough to undergo substitution and thus to study other surface chemistry [26, 28, 29].

Recently, halide‐terminated Ti_3_C_2_Cl_2_ MXenes were reacted with amines to introduce surface amido and imido groups, leading to so‐called hybrid organic–inorganic MXenes (*h*‐MXenes) with a composition of Ti_3_C_2_(NR/NHR)_2/3_ [30]. Solid‐state NMR and density functional theory (DFT) computation showed that amido (NHR) and imido (NR) ligands coexist and occupy the threefold *fcc* hollow sites (μ_3_) at 1/3 coverage in a monodentate fashion. This approach opened a new area of study in integrating the 2D surface chemistry of carbides and nitrides with organic functional groups. As with many synthetically enabling discoveries, it also raises the additional follow‐up questions: (1) Can monolayer coverage be realized? and, (2) Can the amido/imido ligands bind to the surface Ti sites in a bidentate mode? A recent study has demonstrated that the incorporation of ipsilateral diammonium salts with 2D metal halide perovskites can enhance their thermal stability and power conversion efficiency [31]. This observation suggests that there should be similar opportunities in using chelation to drive and control the surface chemistry of *h*‐MXenes. Ti atoms on Ti_3_C_2_ surfaces have a hexagonal close‐packed (*hcp*) structure with the distance between neighboring face‐centered cubic (*fcc*) hollow sites being ∼3.0 Å, which is the ideal size to fit ethylenediamine (en) ligands. Thus, we hypothesize that en‐derived bidentate ligands with minimized steric hindrance can replace labile Br groups on Ti_3_C_2_ surfaces to form stable monolayers.

**FIGURE 1:**
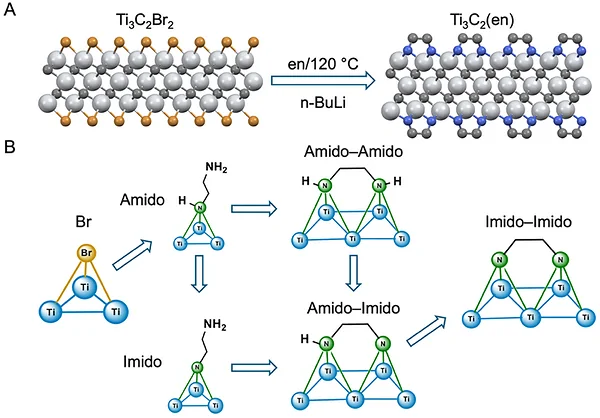
Schematic illustrations: (A) the chelation by ethylenediamine (en) driving the substitution of Br on Ti_3_C_2_Br_2_ to form hybrid MXene Ti_3_C_2_(en), facilitated by *n*‐BuLi to deprotonate en; (B) the substitution and chelation process as the deprotonated en shifts from a monodentate mode (amido or imido) with a free –NH_2_ group to the three bidentate forms of amido–amido, amido–imido, and imido–imido with additional deprotonation. Note that en in Ti_3_C_2_(en) may exist in various deprotonated forms.

This study aims to synthesize and examine the stability and dynamics of en‐derived bidentate binding modes on Ti_3_C_2_ surfaces by substituting Br terminations on Ti_3_C_2_Br_2_. We show that such binding can indeed be achieved on Ti_3_C_2_ surfaces by reacting Ti_3_C_2_Br_2_ with en in strongly basic conditions. The schematic in Figure 1A shows the substitution reaction to reach a monolayer; Figure 1B shows the stepwise process, leading to the three likely modes of bidentate binding from deprotonated en on Ti_3_C_2_: amido–amido (NHC_2_H_4_NH), amido–imido (NHC_2_H_4_N), and imido–imido (NC_2_H_4_N).

## 2 Results and Discussion

**FIGURE 2:**
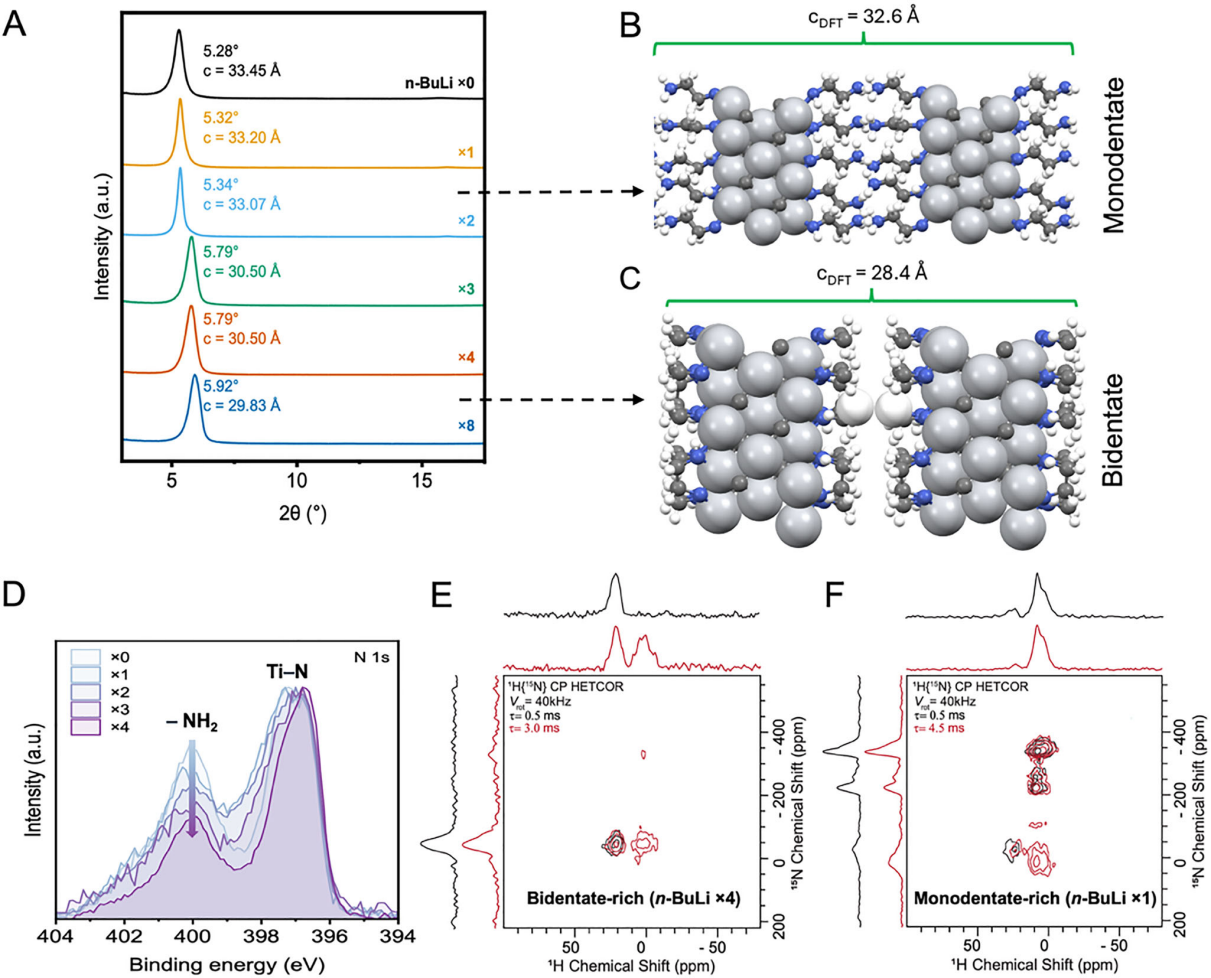
(A) PXRD patterns and the corresponding c lattice parameters of Ti_3_C_2_(en)*_x_* products from reactions of Ti_3_C_2_Br_2_ with ethylenediamine (en) and varying *n*‐BuLi amounts. DFT‐optimized structure and lattice parameter (c_DFT_): (B) Ti_3_C_2_(en_monodent._)_2/3_, comparable with c_exp_ at *n*‐BuLi ×2; (C) Ti_3_C_2_(en_bident._), comparable with c_exp_ at n‐BuLi ×8 (H, white; N, blue; Ti, gray; C, dark gray; two large white spheres showing van der Waals radii of two H atoms in close contact). (D) N 1s XPS spectra of Ti_3_C_2_(en)_x_, showing decreased –NH_2_ component with higher *n*‐BuLi. ^1^H{^15^N} CP‐HETCOR NMR spectra: (E) bidentate‐rich sample (*n*‐BuLi ×4); (F) monodentate‐rich sample (*n*‐BuLi ×1). NMR spectra recorded with a short back CP contact time of 0.5 ms are shown as black traces, while those recorded with longer back CP contact times of 3.0 ms or 4.5 ms are shown as red traces.

The uniqueness of en ligation on Ti_3_C_2_ MXenes, as compared to monoalkylamines, arises from its ability to adopt two distinct bonding configurations: monodentate and bidentate. Figure 2 illustrates the synthetic tunability between these configurations when en substitutes Br on Ti_3_C_2_Br_2_ MXenes. The nearly complete substitution of Br was confirmed by scanning electron microscopy coupled with energy‐dispersive x‐ray spectroscopy (Figure S1). The balance between monodentate and bidentate configurations is dictated by the extent of en deprotonation in the reaction medium: monodentate coordination retains free ─NH_2_ groups, leading to higher protic hydrogen content, whereas bidentate coordination engages both amine termini with a lowered overall protic hydrogen content (Figure 1B). This balance can be precisely regulated by the amount of base (*n*‐BuLi) introduced during synthesis, which directly tunes the monodentate to bidentate ratio in the Ti_3_C_2_(en)*_x_* product and in turn the interlayer spacing. As revealed in the powder x‐ray diffraction (PXRD) patterns (Figures 2A and S2) and scanning tunneling electron microscopy (STEM) images (Figure S3), systematic variations in interlayer spacing were observed with increasing *n*‐BuLi content. Higher base loading decreases the number of protic hydrogen, favoring a bidentate‐dominant configuration characterized by smaller interlayer spacing (c lattice parameter), whereas lower base concentrations preserve monodentate binding and yield larger spacing. DFT relaxations of fully monodentate and fully bidentate Ti_3_C_2_(en)_x_ structures (Figure 2B,C, respectively) show good agreement with the experimental interlayer distances. The interlayer spacing calculated for Ti_3_C_2_(en_monodent._)_2/3_ (32.6 Å) matches the reaction product with lower amounts of *n*‐BuLi (×0, ×1, ×2; 33.45–33.07 Å), while that of Ti_3_C_2_(en_bident._) (28.4 Å) matches the reaction product with higher amounts of *n*‐BuLi (×3, ×4, ×8; 30.5–29.83 Å). The monodentate configuration exhibits interlayer interactions via hydrogen‐bonding between the free –NH_2_ termini (Figure S4), while van der Waals (vdW) interactions between ethylene groups dominate the interlayer interaction in the bidentate configuration (the white spheres in the center of Figure 2C indicate the vdW radii of two H atoms in close contact).

X‐ray photoelectron spectroscopy (XPS) further confirms the shift in the binding mode and the chelation effect (Figure 2D): the N 1s spectra of the Ti_3_C_2_(en)*_x_* products from reaction of Ti_3_C_2_Br_2_ with en and *n*‐BuLi show two bands—at 397 eV corresponding to N in the Ti–N bonding and at 400 eV due to N in the –NH_2_ group. In contrast, the characteristic band of –NH_2_ was not observed from the reaction product of Ti_3_C_2_Br_2_ with monoalkylamine and *n*‐BuLi (Figure S5). The spectra of Ti_3_C_2_(en)*_x_* samples in Figure 2D are normalized to the Ti–N peak, so the decreasing intensity of the –NH_2_ peak with increasing base concentration indicates a transition of deprotonated en from a monodentate binding mode with a terminal –NH_2_ group to a bidentate mode lacking this functionality, as illustrated in Figure 1B.

To further pinpoint the N speciation on the MXene surface, we applied solid‐state NMR (ssNMR) spectroscopy using the ^1^H{^15^N} HETCOR heteronuclear correlation experiments (Figure S6). The ssNMR spectra (Figure 2E,F) show clear differences between the bidentate‐rich sample (*n*‐BuLi ×4) and the monodentate‐rich sample (*n*‐BuLi ×1). In the ^15^N dimension, the bidentate‐rich sample contains two main features (Figure 2E): (1) the resonance near −49 ppm is assigned to surface‐bound amido (–NHR) with a strong correlation with ^1^H NMR signals at a chemical shift of 20 ppm observed with a short contact time, consistent with our previous report [30]; (2) a second, weak feature at −333 ppm is assigned to amino (–NH_2_) groups, consistent with typical amine chemical shifts, which was not observed in our previously reported monoalkylamine‐driven hybrid MXenes [30]. The monodentate‐rich sample shows some of the same features but with different relative intensities (Figure 2F). Specifically, the amino resonance at −350 ppm is substantially more intense, with the amido and imido (═NR) resonances near –30 ppm and 5 ppm, respectively, while the spectrum recorded with a longer contact time of 4.5 ms shows the imido ^15^N NMR signal centered at 5 ppm (Figure S7A), consistent with the fact that these nitrogen atoms do not have an attached ^1^H atom. Integrated peak areas indicate about 80% bidentate mode and 20% monodentate mode in the bidentate‐rich sample (Figure S7B). These results indicate the monodentate‐rich sample prepared at low base loading contains a large fraction of amino groups in addition to surface‐bound amido and imido species, while the very weak amino signal in the bidentate‐rich sample suggests a dominance of the bidentate mode.

**FIGURE 3:**
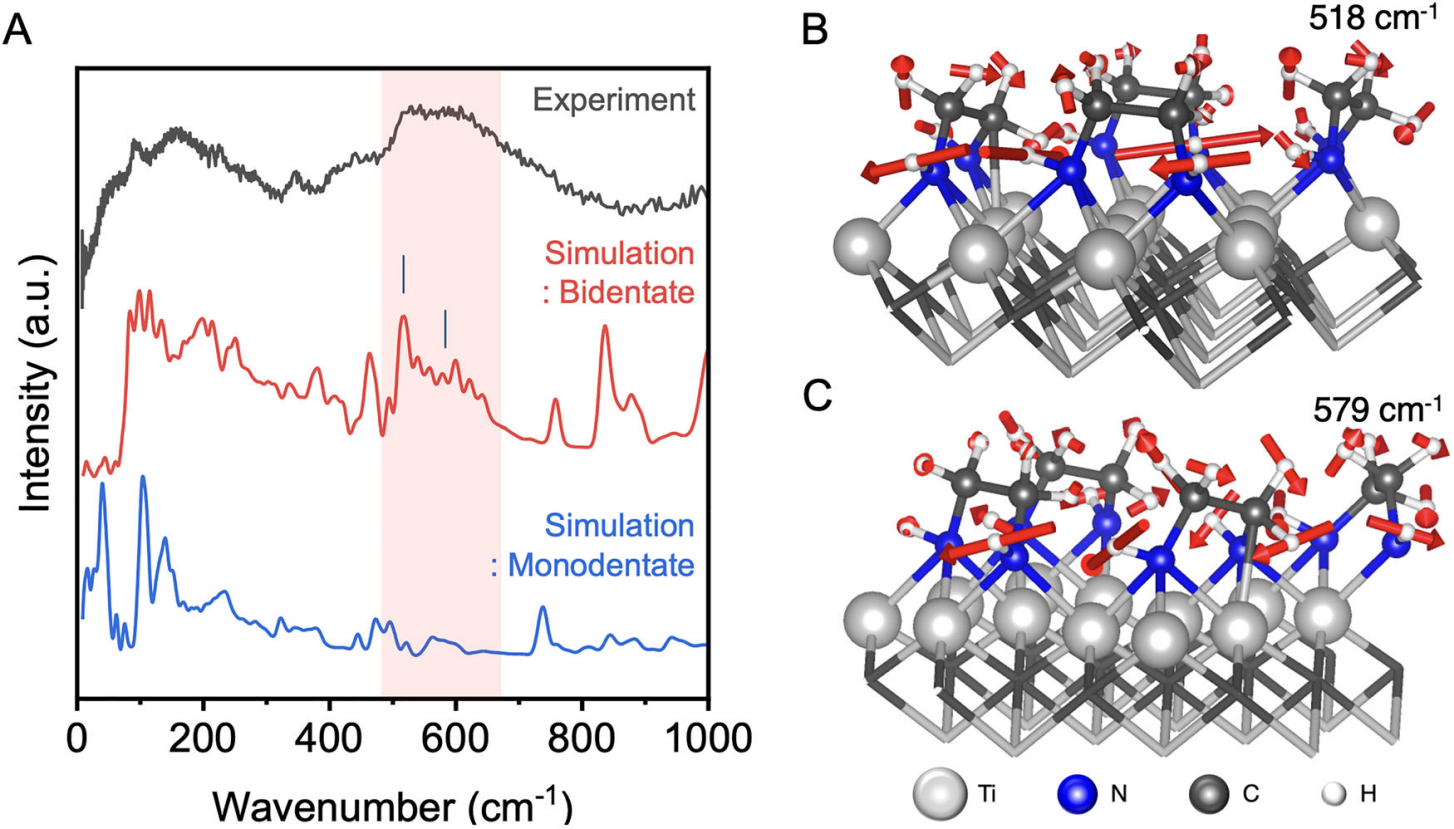
(A) Inelastic neutron scattering (INS) spectra of Ti_3_C_2_(en)*_x_* synthesized by reacting Ti_3_C_2_Br_2_ with en in presence of ×4 *n*‐BuLi, in comparison with simulated INS spectra of Ti_3_C_2_(en_bident._) and Ti_3_C_2_(en_monodent._). The highlighted region indicates the vibrational features of the bidentate binding mode contributed predominately from surface NH or amido groups (two representative modes at the wavenumbers indicated by the two vertical bars are shown in the right panel). (B and C) Representative vibration modes of bidentate en ligands in Ti_3_C_2_(en_bident._) at 518 cm^−1^ and 579 cm^−1^, respectively, in side view.

The metallic nature of Ti_3_C_2_(en)*_x_* makes it difficult to use conventional vibrational spectroscopies to confirm the bidentate binding of deprotonated en moieties on the surface. So we have turned to inelastic neutron scattering (INS), a powerful probe of phonons/vibrations involving hydrogen atoms due to its exceptional sensitivity to hydrogen arising from its orders‐of‐magnitude larger neutron scattering cross section compared to other elements [32]. This selectivity makes INS particularly well‐suited for elucidating the bonding motifs and coordination environments of hydrogen‐containing ligands in solids. Here, we resorted to the VISION spectrometer (see Supporting Information for details), designed for very high neutron flux and energy resolution, particularly in the region below 1000 cm^−1^. Figure 3A shows the INS spectrum of Ti_3_C_2_(en)*_x_* prepared by reacting Ti_3_C_2_Br_2_ with en in the presence of 4 equiv. of *n*‐BuLi, in comparison with simulated INS spectra of Ti_3_C_2_(en_bident._) and Ti_3_C_2_(en_monodent._). The experimental spectrum closely follows the overall spectral features predicted for the bidentate binding model in 0–700 cm^−1^. More importantly, the simulated spectra that incorporate the bidentate mode reproduces the experimentally observed intensity in the 500–650 cm^−1^ region much better than the simulated spectrum of Ti_3_C_2_(en_monodent._). Quantitative analysis of the integrated intensity ratio of I_500–650_/I_0–1000_ confirms a dominant presence of the bidentate mode (0.19) over the monodentate mode (0.08) in comparison with the experimental ratio of 0.21 (Table S1).

Vibrational mode analysis of Ti_3_C_2_(en_bident._) using DFT calculations assigns the feature in the 500–650 cm^−1^ region to collective phonon modes involving mainly N–H vibrations of the amido groups with contributions from C–H vibrations within the –CH_2_–CH_2_– backbone of the bidentate en ligand. Compared to monodentate configuration, the closer spatial proximity and more heterogeneous local environment of N–H bonds in the bidentate motif lead to broadened vibrational features due to the dispersion effect, giving rise to a band rather than discrete peaks in this region. Further analysis reveals that the band is dominated by N–H vibrations (indicated by the long red arrows for two representative modes in Figure 3B,C), with increased contribution from C–H vibrations of the bidentate ligand at higher wavenumbers. The simulated features at 840–900 cm^−1^ of the bidentate mode in Figure 3A arise from the rocking modes of –CH_2_– in the –CH_2_–CH_2_– moieties which are expose to the vacuum in our single‐flake model for Ti_3_C_2_(en_bident._). These modes are expected to be broadened and attenuated in the experimental spectrum due to interlayer interactions in the multilayer sample.

**FIGURE 4:**
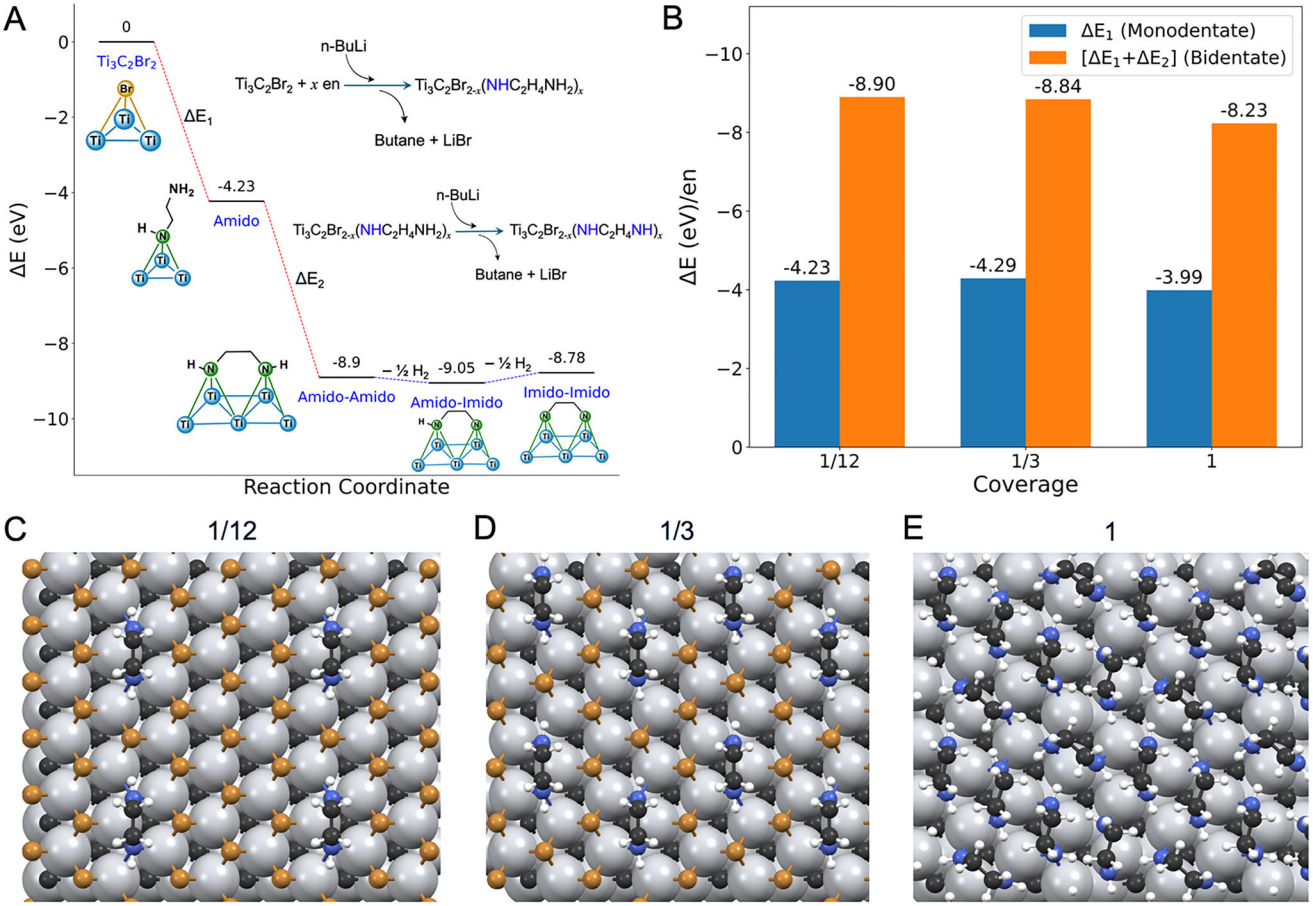
DFT computation of the substitution‐reaction energetics and structure: (A) reaction energy profile of en with Ti_3_C_2_Br_2_ and *n*‐BuLi; (B) coverage‐dependence of the per‐en reaction energy to form Ti_3_C_2_Br_2‐2x_(en)*_x_* where *x* is the en coverage; (C–E) extents of the substitution reactions as indicated by the en coverages at 1/12, 1/3, and 1 (monolayer), respectively (color code: Ti, gray; C, black; Br, brown; N, blue; H, white).

To understand the thermodynamic driving force of the chelate effect of deprotonated en substituting Br on Ti_3_C_2_Br_2_, we computed the reaction energetics (ΔE) of the substitution reactions using DFT to compare the thermodynamic preference of the monodentate vs bidentate configurations. In the experimental conditions during the reaction with Ti_3_C_2_Br_2_, en plays a dual role as both the reactant and the solvent, while the *n*‐BuLi serves as the limiting deprotonation reagent, controlling the extent of deprotonation in the system. Because en is always in large excess, the systems are maintained in a proton‐rich state; increasing the *n*‐BuLi/Ti_3_C_2_Br_2_ ratio promotes more and deeper deprotonation of en, thereby increasing the bidentate fraction. Table S2 lists the proposed stoichiometric reactions leading to full monodentate and bidentate coverages as limiting cases. The energy profile in Figure 4A shows the initiation of the substitution reaction from monodentate substitution of one Br group to bidentate substitution of two Br groups via chelating bis‐amido formation; one sees a downhill trend: the ΔE for –Br to monodentate amido (–NHC_2_H_4_NH_2_) substitution is –4.23 eV (ΔE_1_), which then converts to bidentate amido–amido (–NHC_2_H_4_NH–) with ΔE = –4.67 eV (ΔE_2_). The amido–amido moieties can be further converted to amido–imido (–NHC_2_H_4_N–) with ΔE = –0.15 eV (in reference to ½ H_2_) and then to imido–imido (–NC_2_H_4_N–) with ΔE = 0.27 eV. One can see that the overall reaction is very downhill, showing that the bidentate modes are thermodynamically more favorable. We further compared the monodentate‐amido substitution and the bidentate amido–amido substitution reactions at different coverages up to a monolayer (100% substitution), and one can see the bidentate mode is highly preferred over the whole range (Figure 4B), suggesting minimal steric effects between en ligands with increasing en coverages (Figure 4C–E). At the monolayer coverage, en ligands can pack in different configurations whose relative energies are close based on our sampling of several configurations (Figure S8) due to the limited steric effects; Figure 4E shows one of the more stable bidentate monolayer configurations for Ti_3_C_2_(en) with all amido–amido modes. We note that the DFT energetics shown in Figure 4 do not account for solvation effects, reaction‐environment effects, or entropic contributions, which could influence the relative stability of bidentate vs monodentate configurations. For example, we expect that a protic solvent would stabilize more the monodentate mode where the free –NH_2_ end would have stronger solvation by forming hydrogen bonds with the solvent molecules. Moreover, the proton‐rich reaction environment suggests that proton transfer and dynamics could be important, which is also missing in the DFT energetics but can be explored by ab initio molecular dynamics (AIMD) simulations.

**FIGURE 5:**
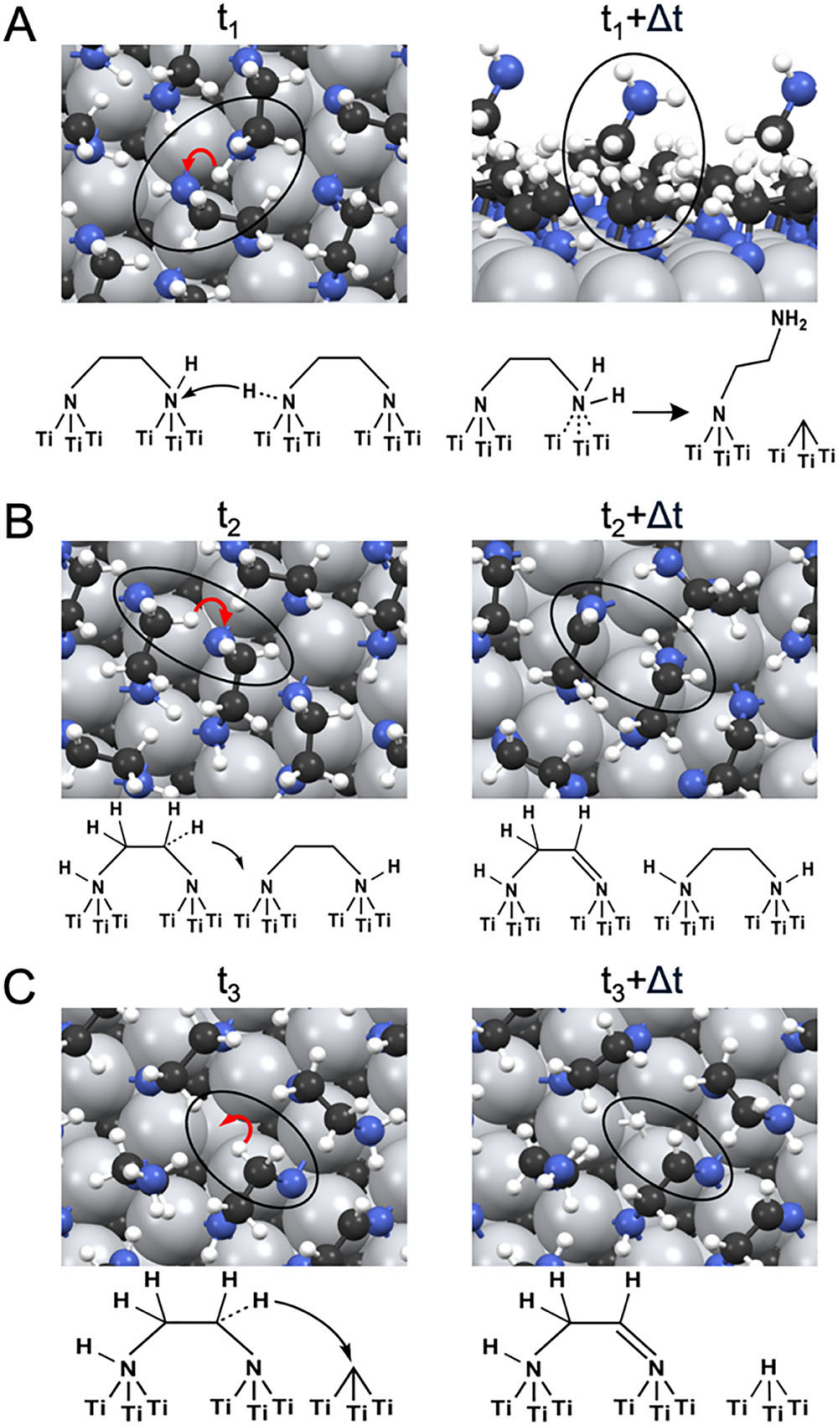
Chemical events observed from the AIMD simulations of Ti_3_C_2_(en) with a monolayer of bidentate en ligands in the amido–imido mode at 600 K: (A) inter‐ligand proton/H transfer between two neighboring amido moieties converting a bidentate ligand to a monodentate ligand; (B) inter‐ligand proton/H transfer between two neighboring imido moieties, leading to a surface imine bond (C═N) and a bidentate amido–amido ligand; (C) formation of surface imine bond from an imido moiety due to β‐H elimination and H transfer to an fcc vacancy to form a surface hydride.

To probe the likely events of proton transfers among the surface‐bound en‐derived ligands, we performed AIMD simulations of Ti_3_C_2_(en) with a monolayer of bidentate en ligands in the amido–imido mode in the NVT ensemble at a moderately higher temperature of 600 K than the experimental reaction temperature of 393 K (120°C), to accelerate the dynamics. We observed inter–ligand proton transfer between two neighboring amido (–NH–) moieties, leading to a switch to the monodentate configuration after the desorption of the –NH_2_ end from the surface (Figure 5A). More interestingly, we found β‐H abstraction from the imido moiety to a neighboring imido group, leading to a surface imine bond (Figure 5B). We also observed imine bond formation when the leaving methylene H migrates to a vacant *fcc* site and forms a surface hydride (Figure 5C). To address whether the observed reactions at 600 K from AIMD persist under synthesis conditions (393 K), we have further used DFT to evaluate the thermodynamics (energy change) and kinetics (activation energies) of those reactions. For the amido‐amido proton transfer and conversion of bidentate to monodentate (Figure 5A), we found an overall barrier of 0.78 eV with Δ*E* = 0.36 eV (Figure S9A), indicating that this reaction is likely to happen kinetically at 393 K though the equilibrium favors the bidentate mode. A simple transition‐state theory estimate (*E*_a_ = 0.78 eV and assuming an attempt frequency of 10^13^ s^−1^) would yield a reaction rate of 10^2^ to 10^3^ s^−1^ per reactive encounter at 393 K. For β‐H abstraction from the imido moiety to a neighboring imido group, leading to a surface imine bond (Figure 5B), we found *E*_a_ = 1.49 eV and Δ*E* = 0.86 eV (Figure S9B), indicating that this reaction is unlikely to happen at 393 K. For β‐H elimination from the imido moiety to an fcc vacancy to form a surface hydride and a surface imine bond (Figure 5C), we found a very low barrier (*E*_a_ = 0.22 eV) with a favorable Δ*E* of –0.21 eV (Figure S9C), indicating that this reaction is very likely to happen at 393 K. Moreover, the combined AIMD and DFT results suggest that the bidentate‐to‐monodentate conversion (Figures 5A and S9A) can be coupled with surface imine bond and hydride formation (Figures 5C and S9C). Such reactions can be more likely in a more realistic partial coverage of both monodentate and bidentate ligands instead of a full coverage of bidentate modes as the starting configuration.

The observation of the surface imine bond in our AIMD simulations of Ti_3_C_2_(en) and confirmed by DFT suggests that it could be an important intermediate in further transformations of the bidentate en ligands on the Ti_3_C_2_ surface. Indeed, the ssNMR spectra revealed a resonance at –220 ppm in the ^15^N dimension of the ^1^H‐detected ^15^N HETCOR spectra (Figure 2F). This feature is consistent with Ti–imine species, based on previously reported ^15^N chemical shifts for molecular Ti–imine coordination complexes [33]. However, we note that the imine assignment remains tentative. A definitive assignment will require additional NMR experiments, potentially including ^13^C isotope labeling. This finding of a surface imine bond also has implications in MXene‐based catalysis because imine bond formation can be a crucial intermediate step in several processes. One example is transition metal‐catalyzed transamination reactions, which create secondary and tertiary amines from primary amines [34] and are also crucial for covalent surface modifications on 2D materials [34, 35].

The monodentate and bidentate Ti_3_C_2_(en)*_*x*_* systems can have different properties and functions. To explore them, we first performed ultraviolet photoelectron spectroscopy (UPS) measurements to determine the work function of Ti_3_C_2_(en)*_x_* samples with different en binding configurations (Figure S10). We found that the bidentate configuration exhibits a work function that is approximately 0.20 eV higher than that of the monodentate configuration (4.86 vs 4.66 eV) in comparison with 5.26 eV for the Ti_3_C_2_Br_2_ precursor. This difference suggests that coordination of both amine termini to the surface partially attenuates the surface dipole induced by en, leading to a less pronounced reduction in work function. The higher work function of the bidentate model is therefore consistent with a more balanced charge distribution and a less polarized interface. Contact‐angle measurements would provide useful probe of surface hydrophobicity/hydrophilicity, but they typically require solution‐processed, continuous films of MXenes. In contrast, the materials studied here are multilayer powders. We attempted contact‐angle measurements on cold‐pressed pellets of our materials, but the water droplets were rapidly absorbed into the porous pellets rather than forming stable sessile droplets. As an alternative, we have computed the theoretical contact angles using Young's law with DFT‐computed surface energies. As shown in Table S3, the monodentate system is predicted to be highly hydrophilic (due to the free –NH_2_ groups), while the bidentate system hydrophobic.

Previous studies established that halogen‐terminated MXenes can undergo covalent surface substitution with monoalkylamines to form alkylamido/imido‐functionalized MXenes, in which the organic groups are attached through a single nitrogen donor and can organize into self‐assembled monolayer‐like interlayer structures [30]. In contrast, the present work introduces en as a chelating diamine ligand, enabling ligand denticity and surface coordination topology to be used as additional synthetic design parameters. Specifically, because en contains two nitrogen donors, it can form both monodentate motifs with pendant amino groups and bidentate motifs in which both nitrogen atoms coordinate to the MXene surface. Importantly, our results show that the relative monodentate/bidentate character can be controlled by the synthetic conditions, particularly the amount of n‐BuLi used during substitution reactions. This capability is distinct from previously reported monoalkylamine‐derived hybrid MXenes, where the ligand is intrinsically monodentate and therefore does not provide access to analogous denticity‐dependent surface configurations. Our work function measurements and computed contact angle results show that controlling the surface coordination configuration of a single ligand can lead to materials with distinct properties. These results highlight ligand denticity, and specifically the monodentate/bidentate balance, as an additional synthetic design parameter for *h*‐MXenes. More broadly, we anticipate this work to motivate further efforts to use surface coordination topology as a materials design principle, where ligands with higher denticity may enable additional structural motifs and emergent properties. We are currently exploring different reaction conditions to control the interlayer/confined reaction kinetics, to push the system toward a full bidentate monolayer with en‐derived ligands. The other idea is to use hindered diamines such as cyclohexanediamines to modulate and prevent the intermolecular proton transfer.

## 3 Conclusions

We have demonstrated *h*‐MXenes functionalized with deprotonated bidentate ethylenediamine (en) ligands by substituting Br on Ti_3_C_2_Br_2_. Controlled deprotonation of –NH_2_ groups using different stoichiometries of *n*‐BuLi drives a transition from monodentate to bidentate binding, evidenced by reduced lattice spacing and decreasing –NH_2_ XPS and NMR signals and supported by DFT geometry optimization. Inelastic neutron scattering confirms bidentate coordination via characteristic vibrational modes of –NH on the surface in the 500–650 cm^−1^ region. DFT calculations show that bidentate binding is significantly more stable than monodentate modes and can fully replace Br terminations; on the other hand, ab initio molecular dynamics simulations of the Ti_3_C_2_(en) monolayer of amido–imido en ligands reveal rich and facile inter–ligand proton transfer and back‐conversion of bidentate to monodentate modes as well as surface imine bond formation. This work demonstrates how ligand denticity enables new surface chemistry in *h*‐MXenes, with implications for MXene properties and functions.

## Conflicts of Interest

The authors declare no conflicts of interest.

## Supporting information




**Supporting File**: Supporting information includes experimental and computational details and additional data. The authors have cited additional references within the Supporting Information [36, 37, 38, 39, 40, 41, 42, 43, 44, 45, 46, 47, 48, 49, 50, 51, 52, 53, 54, 55, 56, 57, 58].

## Data Availability

The data that support the findings of this study are available from the Zenodo repository (https://doi.org/10.5281/zenodo.21314669) as well as from the corresponding authors upon reasonable request.
