# Supplementary material for: Chelation Drives Surface Substitution in Hybrid‐MXenes
Source: Angew Chem Int Ed Engl. 2026 Jul 20;65(37):e6432447. doi: 10.1002/anie.6432447 (PMC13549004; doi:10.1002/anie.6432447)
Supplement: Supplementary file 1 [file ANIE-65-e6432447-s001.docx]

Supporting Information

**Chelation Drives Surface Substitution in Hybrid-MXenes**

Vikash Khokhar^#[a]^, Young-Hwan Kim^#[b]^, Abu Rashed Md. Shawon^[c,d]^, Fatemeh Karimi^[e]^, Danial Zangeneh^[e]^, Yongqiang Cheng^[f]^, Chang Liu^[g]^, Murillo Longo Martins^[f]^, Alexander S. Filatov^[b]^, Robert F. Klie^[e]^, Aaron J. Rossini*^[c,d]^, John S. Anderson*^[b]^, Dmitri V. Talapin*^[b]^, De-en Jiang*^[h]^

[a]	V. Khokhar

Interdisciplinary Materials Science, Vanderbilt University, Nashville, Tennessee 37235, United States

[b]	Y. H. Kim, Dr. A. S. Filatov, Prof. Dr. J. S. Anderson, Prof. Dr. D. V. Talapin

Department of Chemistry, Pritzker School of Molecular Engineering, and James Franck Institute, University of Chicago, Chicago, Illinois 60637, United States

E-mail: [jsanderson@uchicago.edu](mailto:jsanderson@uchicago.edu); [dvtalapin@uchicago.edu](mailto:dvtalapin@uchicago.edu)

[c]	A. R. Md. Shawon, Prof. Dr. A.J. Rossini

Division of Materials Science and Engineering, Ames National Laboratory, Ames, Iowa 50011, United States

E-mail: [arossini@iastate.edu](mailto:arossini@iastate.edu)

[d] A. R. Md. Shawon, Prof. Dr. A.J. Rossini

Department of Chemistry, Iowa State University, Ames, Iowa 50011, United States

[e]	F. Karimi, D. Zangeneh, Prof. Dr. R. F. Klie

Department of Physics, University of Illinois Chicago, Chicago, Illinois 60607, United States

[f]	Dr. Y. Cheng, Dr. M. L. Martins

Neutron scattering division, Oak Ridge National Laboratory, Oak Ridge, Tennessee 37831, United States

[g]	Dr. C. Liu

Chemical science division, Oak Ridge National Laboratory, Oak Ridge, Tennessee 37831, United States

[h]	Prof. Dr. D. Jiang

Department of Chemical and Biomolecular Engineering, Vanderbilt University, Nashville, Tennessee 37235, United States

E-mail: [de-en.jiang@vanderbilt.edu](mailto:de-en.jiang@vanderbilt.edu)

[#]	These authors contributed equally to the work.

**Materials and Methods**

Chemicals

The following chemicals were used without further purification: toluene (99.8%, anhydrous), methanol (99.8%, anhydrous), *n*-butyllithium (*n*-BuLi, 2.5 M in hexanes) were from Sigma-Aldrich; Ti powder (99.5%, 325 mesh), Al powder (99.5%, 325 mesh), graphite (C, natural, 99.8%, 325 mesh) were from Alfa Aesar; cadmium chloride (CdCl_2_, 99+%, anhydrous), cadmium bromide (CdBr_2_, 99%, anhydrous) were from Strem Chemicals; ethylenediamine (98.0+%, anhydrous) was from TCI America.

The following chemicals were distilled over sodium and stored inside an inert atmosphere glovebox: propylamine (≥99%), octylamine (99%), and dodecylamine (98%) were purchased from Sigma-Aldrich.

The following chemicals were degassed and dried over molecular sieves and stored inside an inert atmosphere glovebox: ^15^N-ethylenediamine (99%) was purchased from ICON isotopes.

Synthesis of Ti_3_AlC_2_ MAX phase

Ti_3_AlC_2_ MAX phase was synthesized using a modified literature procedure.^[1]^ In brief, Ti (3.661 g), C (0.566 g), and Al (0.757 g) powders (3:1.85:1.1 molar ratio) were mixed and cold pressed into a pellet. The resulting pellet was placed in an alumina crucible and heated at 1650 °C for 6 h under flowing Ar. The resulting product was ground using a mortar and pestle and sieved to 450 mesh prior to further use.

Synthesis of Cl- and Br- terminated MXenes

Halogen-terminated Ti_3_C_2_ MXenes were synthesized using a modified literature procedure.^[1,2]^ Ti_3_AlC_2_ (0.1–3.0 g) MAX phase was mixed with CdCl_2_ or CdBr_2_ salts in a 1:8 molar ratio and transferred to an alumina or silicon nitride crucible. The mixture was heated under Ar at 610 °C for at least 6.5 h. For Cl-terminated MXenes, the reaction product was treated with concentrated aqueous HCl (37%) to dissolve excess CdCl_2_ and metallic Cd, followed by repeated washing with deionized water until the pH of washing was neutral. For Br-terminated MXenes, excess CdBr_2_ and metallic Cd were removed using concentrated aqueous HBr (48%), followed by similar aqueous washing using deionized water. The resulting halogen-terminated MXene powders were dried under vacuum at 40 °C for >12 h prior to further use.

Synthesis of hybrid organic-inorganic MXenes

Synthesis of hybrid organic-inorganic MXenes were performed using a modified literature procedure.^[2]^ All reactions were performed using an inert atmosphere using an N_2_-filled glovebox with oxygen and moisture levels below 0.1 ppm. In a typical procedure, halogen-terminated MXene powders were combined with the desired amine, and a specific amount of *n*-BuLi was added. The mixture was stirred with a glass stir bar at 120°C in a heavy-walled glassware for a defined duration. After completion, the products were washed with methanol and dried under vacuum prior to further use.

X-ray diffraction (XRD)

Powder X-ray diffraction patterns in reflection mode were collected using a Rigaku benchtop diffractometer equipped with a HyPix-400 MF two-dimensional hybrid pixel array detector (HPAD) and a Cu Kα radiation source (λ = 1.5418 Å), operated at 40 kV and 15 mA.

X-ray photoelectron spectroscopy (XPS)

XPS measurements were performed on a Kratos Axis Nova spectrometer equipped with a monochromatic Al Kα source (1486.6 eV). High-resolution spectra were recorded with a pass energy of 20 eV, step size of 100 meV, and an analysis area of 0.3 × 0.7 mm². Charge neutralization was achieved using a co-axial, low-energy (≈ 0.1 eV) electron flood gun to avoid shifts in the recovered binding energy.

Solid-state nuclear magnetic resonance (ssNMR) spectroscopy

Solid-state NMR spectroscopy experiments were performed on a Bruker wide-bore 9.4 T [*ν*_0_(^1^H) = 400 MHz] NMR spectrometer equipped with a Bruker AVANCE III HD console. All experiments were performed with a Bruker 1.3 mm HX double resonance probe. Samples were packed into 1.3 mm zirconia rotors in a dry nitrogen glovebox. All experiments were performed with a 40 kHz MAS frequency. The MAS frequency was limited to 40 kHz due to increased drive pressures required to spin these samples above 40 kHz. The resistance to sample spinning presumably arose from the metallic nature of the samples. Furthermore, we also observed that the MXene samples partially absorbed the RF power from the pulses or shielded themselves from the RF pulses. Therefore the ^1^H and ^15^N RF fields were directly calibrated on each sample. ^1^H RF fields were calibrated by observing nutation profiles using a pulse-acquire sequence and varying the duration of the excitation pulse. The ^1^H RF field was then confirmed by using a spin-lock pulse sequence to observe rotary resonance conditions (n_1_ = 0.5 × n_rot_). ^15^N RF fields were approximately estimated by varying the p/2 pulse durations within the ^1^H-detected ^15^N CP-HETCOR NMR experiment. ^1^H→^15^N cross-polarization (CP) conditions were directly optimized on each sample. ^1^H saturation recovery NMR experiment were used to measure the ^1^H longitudinal relaxation time constant (*T*_1_). ^1^H *T*_1_ of 1.15 s and 1.7 s were measured for monodentate-rich and bidentate-rich Ti_3_C_2_(en)*_x_* samples, consequently, recycle delays of 1.5 s and 2.21 s were used for all subsequent NMR experiments, respectively. These recycle delays correspond to approximately 1.3×*T*_1_ and were used to maximize sensitivity. 2D ^1^H{^15^N} HETCOR^[3–6]^ NMR experiments on the MXene with monodentate en were performed with a forwards CP contact time of 4.5 ms and a long back CP contact time of 4.5 ms or a short back CP contact time of 0.5 ms. 2D ^1^H{^15^N} HETCOR NMR experiments on the bidentate-rich sample were performed with a 4 ms forwards CP contact time and a long back CP contact time of 3 ms or a short back CP contact time of 0.5 ms. Indirect ^15^N dimension was achieved with the STATES-TPPI method.^[7]^ 640 scans were acquired for each *t*_1_-increment. The *t*_1_-evolution time was incremented in steps of 25 µs and 80 *t*_1_-increments were acquired for both MXenes. The ^1^H→^15^N CP transfers on the bidentate-rich sample used ^1^H and ^15^N spin-lock RF fields of 70 kHz and 32 kHz. The ^1^H→^15^N CP transfers on the monodentate-rich sample used ^1^H and ^15^N spin-lock RF fields of 88 kHz and 54 kHz. The amplitude of the 1H RF field during CP spin-lock pulses was sampled from 90% to 100% of the indicated values to broaden the CP match condition. All NMR spectra were processed in TopSpin 3.8.0 software. ^1^H chemical shifts were referenced to neat tetramethylsilane using adamantane (*δ*_iso_ (^1^H) = 1.82 ppm) as a secondary chemical shift reference. ^15^N chemical shifts were indirectly referenced to neat MeNO_2_ (*δ*_iso_ = 0 ppm) using the IUPAC recommended Larmor frequency^[8]^ ratio of 10.136767%.

Scanning electron microscopy-energy dispersive X-ray spectroscopy (SEM-EDS)

SEM imaging and EDS elemental mapping were performed in a Carl Zeiss Merlin Field-Emission Scanning Electron Microscope equipped with Oxford Ultim Max 100 Silicon Drift Detectors (SDD). The accelerating voltage and beam current was set to 10 kV and 1 nA, respectively.

Scanning transmission electron microscopy (STEM)

*h*-MXene samples were characterized using an aberration-corrected JEOL ARM200CF scanning transmission electron microscope (STEM) at the University of Illinois Chicago. The microscope operates at 200 kV and is equipped with a cold field emission gun and a Gatan Continuum electron energy-loss spectroscopy (EELS) system, enabling a sub-angstrom probe size and an energy resolution of approximately 350 meV. To limit beam-induced damage, the emission current was set to 12 μA. Imaging was carried out with a probe convergence semi-angle of 24 mrad. High-angle annular dark-field (HAADF) imaging employed detector angles ranging from 90 to 370 mrad, while low-angle annular dark-field (LAADF) imaging used a range of 40 to 160 mrad. EELS data were acquired using a post-column Gatan Continuum Imaging Filter, with a probe convergence semi-angle of 17.8 mrad and a collection angle of 53.4 mrad. A Gatan 636 double-tilt liquid nitrogen holder, capable of cooling down to −170 °C while preserving atomic resolution, was utilized to mitigate beam damage during measurements.

Inelastic neutron scattering (INS)

The inelastic neutron scattering results presented in this work were collected at VISION. VISION is a backscattering spectrometer at the pulsed spallation neutron source at the Oak Ridge National Laboratory. At this instrument, the sample is exposed to a white neutron beam and the final energy of the scattered neutrons is fixed by Bragg reflection on pyrolytic graphite analyzers. All the samples were loaded in vanadium cans (6 mm) and then measured at ~ 10 K. Data was reduced using the software Mantid.^[9]^

Vibrational spectra of the DFT relaxed configurations were calculated using the finite displacement method with Phonopy.^[10]^ The calculated eigen-frequencies and eigen-modes on a 5x5x1 grid in the Brillouin zone were then used as input of OCLIMAX^[11]^ to generate simulated VISION spectra for the DFT relaxed configurations.

To quantitatively compare the intensities for monodentate vs bidentate configurations with experimental intensities, we calculated the integrated normalized intensity (Area) ratio in the diagnostic region 500-650 cm^-1^. The intensities were integrated using the trapezoidal rule. The integrated intensity ratio (I_500-650_/I_0-1000_) was then compared for monodentate, bidentate, and experimental spectra.

Ultraviolet photoelectron spectroscopy (UPS)

UPS measurements were performed on a Kratos Axis Nova spectrometer using He I emission line (21.22 eV) as the radiation source. Samples were prepared as cold-pressed pellets with a diameter of 1 cm. A bias of −9 V was applied between the sample and the analyzer during the measurements. The energy resolution was 100 meV. Prior to measurement, the measurement area was gently cleaned using Ar_2000_^+^ clusters at 5 keV for 2 min to remove native surface TiO_2_ formed during air exposure associated with sample transfer, which has been shown to remove surface and edge oxides without disturbing the MXene surface.^[2]^

Density functional theory (DFT) calculations and *ab initio* MD simulations

Density functional theory (DFT) calculations were performed with the Vienna *Ab initio* Simulation Package (VASP) software package from the University of Vienna.^[12]^ The PBE exchange-correlation functional proposed by Perdew, Burke, and Ernzerhof was used with projector-augmented-wave (PAW) pseudopotentials.^[13–15]^ The 500 eV plane wave cutoff energy was used, and atomic positions were relaxed until the force convergence of 0.01 eVÅ^-1^. DFT-D3 method was applied to account for the dispersion corrections.^[16]^ Monkhorst-Pack k-points grid 3x3x1 sampled the Brillouin zone.^[17]^ Nudged elastic band (NEB) calculations were done using the climbing image method to calculate the barriers with VTST tools.^[18–20]^

For *ab initio* MD simulations (AIMD)^[21]^, Nose-Hoover thermostat^[22]^ with standard molecular dynamics in NVT ensemble was implemented with a time-step of 1 fs. The simulation was run for 10 ps, keeping initial velocities constant at a 600 K temperature.

The Ti_3_C_2_ MXene surface, with varying Br and en terminations, was constructed as a supercell of 12 units of Ti_3_C_2_, with lattice parameters 10.45x9.04 Å, as used for *h-*MXenes and a large vacuum in the Z-direction.^[2]^ Full coverage of en-derived bidentate ligand structure was geometry optimized and utilized for AIMD simulation.

The reaction energetics (ΔE) for the following reactions were calculated to compare the thermodynamic preference of the monodentate vs bidentate configurations (Figure 4).

For monodentate configuration,

12 Ti_3_C_2_Br_2_ + n NH_2_C_2_H_4_NH_2_ (g) + n (n-BuLi) (g) → 12 Ti_3_C_2_Br_x_(NHC_2_H_4_NH_2_)_y_ + n Butane (g) + n LiBr (s)

$$\Delta E=\frac{\begin{aligned} \left( E\left[ 12Ti_{3}C_{2}{Br}_{x}{{(NH}_{2}C_{2}H_{4}{NH}_{2})}_{y} \right]+n\times E\left[ \mathrm{Butane} \right]+n\times E\left[ LiBr \right] \right) \\ -\left( E\left[ 12Ti_{3}C_{2}{Br}_{2} \right]+n\times E\left[ {NH}_{2}C_{2}H_{4}{NH}_{2} \right]+n\times E\left[ n-BuLi \right] \right) \end{aligned}}{n}$$

For bidentate configuration,

12 Ti_3_C_2_Br_2_ + n NH_2_C_2_H_4_NH_2_ (g) + 2n (n-BuLi) (g) → 12 Ti_3_C_2_Br_x_(NHC_2_H_4_NH)_y_ + 2n Butane (g) + 2n LiBr (s)

$$\Delta E=\frac{\begin{aligned} \left( E\left[ 12Ti_{3}C_{2}{Br}_{x}{(NHC_{2}H_{4}\mathrm{NH})}_{y} \right]+2n\times E\left[ \mathrm{Butane} \right]+2n\times E\left[ LiBr \right] \right) \\ -\left( E\left[ 12Ti_{3}C_{2}{Br}_{2} \right]+n\times E\left[ {NH}_{2}C_{2}H_{4}{NH}_{2} \right]+2n\times E\left[ n-BuLi \right] \right) \end{aligned}}{n}$$

Full coverages of the three bidentate ligand modes were geometry-optimized, and the binding energy was computed according to the equation below, where $E\left[ 12Ti_{3}C_{2} \right]$, $E\left[ Ligand \right]$, and $E\left[ 12Ti_{3}C_{2}\left( Ligand \right) \right]$ are the energies of termination-free MXene, free ligand in the gas phase, and fully ligand-covered MXene with 12 ligands (Figure S8).

$$E_{BE}=\frac{\left( E\left[ 12Ti_{3}C_{2} \right]+12\times E\left[ Ligand \right]-E\left[ 12Ti_{3}C_{2}\left( Ligand \right) \right] \right)}{12}$$

DFT+AIMD-based Young’s law calculations were performed to estimate the intrinsic wettability of MXene surfaces functionalized with monodentate and bidentate en ligands (Ti_3_C_2_(en_mono_)_2/3_ and Ti_3_C_2_(en_bi_)). AIMD simulations were carried out in the NVT ensemble at 300 K for 5 ps for MXene/H_2_O interfacial systems with a 12 Å water layer, as well as for the corresponding water-only reference system, to obtain equilibrated water structures and realistic interfacial configurations. In our previous study, we reported that water-MXene interface systems can be equilibrated using short AIMD simulations.^[23]^ The equilibrated 10 AIMD snapshots were subsequently geometry-optimized for both systems using the non-local vdW-DF dispersion-correlation functional to account for complex interfacial van der Waals interactions ^[24,25]^ and obtain consistent DFT energy averages. Additional DFT optimizations were performed for the bulk MXene reference and the dry MXene slab in vacuum. The resulting energies were used to calculate the solid–vapor ($\gamma_{SV}$), solid–liquid ($\gamma_{SL}$), and liquid–vapor ($\gamma_{LV}$) interfacial energies, which were then substituted into Young’s law equation, to estimate the water contact angle and compare the effect of monodentate versus bidentate ligand binding on MXene wettability. A in the formulas is the lateral surface area of the simulation cell, and the factor of 2 accounts for the two equivalent interfaces of each system.

Young's law:

$$\gamma_{SV}=\gamma_{SL}+\gamma_{LV}\cos\theta$$

$$\cos\theta=\frac{\gamma_{SV}-\gamma_{SL}}{\gamma_{LV}}$$

And the interfacial energies are calculated by the following.

$$\gamma_{SV}=\frac{E_{MX layler}-E_{MX \mathrm{bulk}}}{2A}$$

$$\gamma_{SL}=\frac{E_{\mathrm{MX}\mathrm{layer}+H_{2}O}-E_{MX \mathrm{bulk}}-E_{H_{2}O bulk}}{2A}$$

$$\gamma_{LV}=\frac{E_{H_{2}O layer}-E_{H_{2}O bulk}}{2A}$$

Figure S1. (A) The amount of Br with respect to Ti (measured by SEM-EDS) after reacting Ti_3_C_2_Br_2_ with ethylenediamine for 48 h using varying amounts of *n*-BuLi. (B) SEM-EDS elemental mapping of Ti_3_C_2_(en)_x_ synthesized with *n*-BuLi ×4. Both results confirm substitution of Br with en-derived motifs.

Figure S2. Powder XRD patterns of Ti_3_AlC_2_ MAX phase, Ti_3_C_2_Br_2_ MXenes, and Ti_3_C_2_(en)*_x_* MXenes. Detailed synthetic procedures for each material are provided in the Materials and Methods section.

Figure S3. Annular dark-field (ADF) STEM images of Ti_3_C_2_(en)_x_ synthesized by reacting Ti_3_C_2_Br_2_ with en in the presence of (A) ×4 *n*-BuLi and (B) ×1 *n*-BuLi. Two images are shown at the same magnification to highlight the difference in interlayer spacing. Corresponding selected-area electron diffraction (SAED) patterns for the (C) ×4 *n*-BuLi and (D) ×1 *n*-BuLi samples further confirm the difference in interlayer spacing.

Figure S4. DFT-optimized interlayer spacing structure of Ti_3_C_2_(en_monodent._)_2/3_ showing interlayer interactions through hydrogen bonding between the free amine termini (–NH_2_).

Figure S5. N 1s XPS of Ti_3_C_2_(oca)_2/3_ MXenes, showing only the Ti–N component (397 eV) and lacking the –NH_2_ feature (~400 eV) observed in Ti_3_C_2_(en)*_x_* MXenes.

Figure S6. Pulse sequence used for ^1^H-detected ^15^N CP-HETCOR experiments on Ti_3_C_2_(en)*_x_* MXenes.

Figure S7. ssNMR spectra of Ti_3_C_2_(en)_x_ samples. (A) ^15^N ssNMR spectra of the monodentate-rich sample extracted from summing the columns of the ^1^H{^15^N} CP-HETCOR NMR spectra. NMR spectra obtained with back CP contact times of 0.5 ms and 4.5 ms are shown. The longer contact time spectrum will give nearly quantitative ^15^N NMR peak intensities, while the spectrum recorded with a short contact time will exclusively show NMR signals from protonated nitrogen atoms. Fits of the amido and imido peaks to Voigt peaks are shown, with relative peak areas indicated. (B) ^15^N ssNMR spectra of the monodentate-rich and bidentate-rich samples obtained by summing the columns of the ^1^H{^15^N} CP-HETCOR NMR spectra with the indicated back-CP contact times. Peak integrations are shown for the ^15^N ssNMR spectra recorded with longer back CP contact times for both the monodentate-rich (top) and bidentate-rich (bottom) samples of Ti_3_C_2_(en)_x_. Relative integrated intensities are indicated for each integrated region.

Figure S8. DFT-optimized monolayer Ti_3_C_2_(en) structures with bidentate en ligands of different packing configuration: (A) structure-1 (high translational symmetry); (B) structure-2 (medium C_2_ symmetry); (C) structure-3 (medium C_2_ symmetry); (D) structure-4 (low symmetry, no point-group). Average binding energy per ligand for four different monolayer structures on Ti_3_C_2_ surface: (E) amido–amido; (F) amido–imido; (G) imido–imido. Color code: Ti, gray; N, blue; –CH_2_–CH_2_– group, dark gray line).

Figure S9. DFT-computed minimum-energy paths for the chemical events observed from the AIMD simulations of Ti_3_C_2_(en) with a monolayer of bidentate en ligands in the amido–imido mode at 600 K: (A) proton transfer between two bidentate amido–imido ligands from amido to amido and then the bidentate ligand converting to a monodentate ligand; (B) β-H elimination and H transfer to neighbouring imido moiety, leading to a surface imine bond (C=N) and a bidentate amido–amido ligand; (C) formation of surface imine bond from an imido moiety due to β-H elimination and H transfer to an fcc vacancy to form a surface hydride.

Figure S10. **Ultraviolet photoelectron spectroscopy (UPS) measurement of work-function.** (A) Secondary-electron cutoff regions of Ti_3_C_2_Br_2_ and Ti_3_C_2_(en)_x_ at x1 n-BuLi (monodentate-rich) and x8 n-BuLi (bidentate-rich) samples. (B, C) Linear extrapolation of the secondary-electron cutoff used to determine the work functions of Ti_3_C_2_Br_2_ and Ti_3_C_2_(en)_x_ samples. (D) Extracted work-function values showing a systematic decrease from Ti_3_C_2_Br_2_ to bidentate-rich and monodentate-rich Ti_3_C_2_(en)_x_.

Table S1. Integrated INS intensity ratios for the experimental spectrum of Ti₃C₂(en)_x_ (x4 eq n‑BuLi) and simulated spectra of Ti₃C₂(en_bident._) and Ti₃C₂(en_monodent._).

| INS spectrum | I_500–650_/I_0–1000_ |
| --- | --- |
| Experiment | 0.21 |
| Simulation: Bidentate | 0.19 |
| Simulation: Monodentate | 0.08 |

Table S2. Proposed stoichiometric reactions of Ti_3_C_2_Br_2_, n-BuLi, and en

| en mode | Proposed reactions |
| --- | --- |
| monodentate | 1 equiv. n-BuLi to Ti_3_C_2_Br_2_  Ti_3_C_2_Br_2_ + n-BuLi + en → Ti_3_C_2_Br(en_amido-amino_) + LiBr + Butane |
|  | 2 equiv. n-BuLi to Ti_3_C_2_Br_2_  Ti_3_C_2_Br_2_ + 2n-BuLi + 2en → Ti_3_C_2_(en_amido-amino_)_2_ + 2LiBr + 2Butane |
| bidentate | 3 equiv. n-BuLi to Ti_3_C_2_Br_2_  Ti_3_C_2_Br_2_ + 3n-BuLi + 2en → Ti_3_C_2_(en_amido-amido_) + Li(en_amido-amino_) + 2LiBr + 3Butane |
|  | 4 equiv. n-BuLi to Ti_3_C_2_Br_2_  Ti_3_C_2_Br_2_ + 4n-BuLi + 3en → Ti_3_C_2_(en_amido-amido_) + 2Li(en_amido-amino_) + 2LiBr + 4Butane |

Table S3. Estimated contact angle (θ) for monodentate and bidentate models of Ti_3_C_2_(en)_x_ using Young’s Law and DFT-calculated interfacial energies: solid-vapor (γ_SV_); solid-liquid (γ_SL_); liquid-vapor (γ_LV_).

| System | γ_SV_ (J/m^2^) | γ_SL_ (J/m^2^) | γ_LV_ (J/m^2^) | Contact angle (θ) |
| --- | --- | --- | --- | --- |
| Ti_3_C_2_(en_monodent._)_2/3_ | 0.115 | −0.108 ± 0.023 | 0.112 ± 0.016 | ~0° |
| Ti_3_C_2_(en_bident._) | 0.133 | 0.134 ± 0.021 | 0.112 ± 0.016 | ~90.4° ± 10.8° |

**References**

[1]	V. Kamysbayev, A. S. Filatov, H. Hu, X. Rui, F. Lagunas, D. Wang, R. F. Klie, D. V. Talapin, “Covalent surface modifications and superconductivity of two-dimensional metal carbide MXenes” *Science* **2020**, *369*, 979–983. <https://doi.org/10.1126/science.aba8311>.

[2]	C. Zhou, D. Wang, F. Lagunas, B. Atterberry, M. Lei, H. Hu, Z. Zhou, A. S. Filatov, D. Jiang, A. J. Rossini, R. F. Klie, D. V. Talapin, “Hybrid organic–inorganic two-dimensional metal carbide MXenes with amido- and imido-terminated surfaces” *Nat. Chem.* **2023**, *15*, 1722–1729. <https://doi.org/10.1038/s41557-023-01288-w>.

[3]	A. Venkatesh, M. J. Ryan, A. Biswas, K. C. Boteju, A. D. Sadow, A. J. Rossini, “Enhancing the Sensitivity of Solid-State NMR Experiments with Very Low Gyromagnetic Ratio Nuclei with Fast Magic Angle Spinning and Proton Detection” *J. Phys. Chem. A* **2018**, *122*, 5635–5643. <https://doi.org/10.1021/acs.jpca.8b05107>.

[4]	Z. Wang, M. P. Hanrahan, T. Kobayashi, F. A. Perras, Y. Chen, F. Engelke, C. Reiter, A. Purea, A. J. Rossini, M. Pruski, “Combining fast magic angle spinning dynamic nuclear polarization with indirect detection to further enhance the sensitivity of solid-state NMR spectroscopy” *Solid State Nucl. Magn. Reson.* **2020**, *109*, 101685. <https://doi.org/10.1016/j.ssnmr.2020.101685>.

[5]	Y. Ishii, R. Tycko, “Sensitivity Enhancement in Solid State 15N NMR by Indirect Detection with High-Speed Magic Angle Spinning” *J. Magn. Reson.* **2000**, *142*, 199–204. <https://doi.org/10.1006/jmre.1999.1976>

[6]	J. W. Wiench, C. E. Bronnimann, V. S.-Y. Lin, M. Pruski, “Chemical Shift Correlation NMR Spectroscopy with Indirect Detection in Fast Rotating Solids: Studies of Organically Functionalized Mesoporous Silicas” *J. Am. Chem. Soc.* **2007**, *129*, 12076–12077. <https://doi.org/10.1021/ja074746+>.

[7]	D. Marion, M. Ikura, R. Tschudin, A. Bax, “Rapid recording of 2D NMR spectra without phase cycling. Application to the study of hydrogen exchange in proteins” *J. Magn. Reson.* *(1969)* **1989**, *85*, 393–399. <https://doi.org/10.1016/0022-2364(89)90152-2>

[8]	R. K. Harris, E. D. Becker, S. M. Cabral De Menezes, R. Goodfellow, P. Granger, “NMR Nomenclature: Nuclear Spin Properties and Conventions for Chemical Shifts” *Solid State Nucl. Magn. Reson.* **2002**, *22*, 458–483. <https://doi.org/10.1006/snmr.2002.0063>

[9]	O. Arnold, J. C. Bilheux, J. M. Borreguero, A. Buts, S. I. Campbell, L. Chapon, M. Doucet, N. Draper, R. Ferraz Leal, M. A. Gigg, et al., “Mantid—Data analysis and visualization package for neutron scattering and μ SR experiments” *Nucl. Instrum. Methods Phys. Res. A* **2014**, *764*, 156–166. <https://doi.org/10.1016/j.nima.2014.07.029>.

[10]	A. Togo, L. Chaput, T. Tadano, I. Tanaka, “Implementation strategies in phonopy and phono3py” *J. Phys.: Condens. Matter.* **2023**, *35*, 353001. <https://doi.org/10.1088/1361-648X/acd831>.

[11]	Y. Q. Cheng, L. L. Daemen, A. I. Kolesnikov, A. J. Ramirez-Cuesta, “Simulation of Inelastic Neutron Scattering Spectra Using OCLIMAX” *J. Chem. Theory Comput.* **2019**, *15*, 1974–1982. <https://doi.org/10.1021/acs.jctc.8b01250>.

[12]	G. Kresse, J. Furthmüller, “Efficiency of ab-initio total energy calculations for metals and semiconductors using a plane-wave basis set” *Comp. Mater. Sc.* **1996**, *6*, 15–50. <https://doi.org/10.1016/0927-0256(96)00008-0>.

[13]	J. P. Perdew, K. Burke, M. Ernzerhof, “Generalized Gradient Approximation Made Simple” *Phys. Rev. Lett.* **1996**, *77*, 3865–3868. <https://doi.org/10.1103/PhysRevLett.77.3865>.

[14]	P. E. Blöchl, “Projector augmented-wave method” *Phys. Rev. B* **1994**, *50*, 17953–17979. <https://doi.org/10.1103/PhysRevB.50.17953>.

[15]	G. Kresse, D. Joubert, “From ultrasoft pseudopotentials to the projector augmented-wave method” *Phys. Rev. B* **1999**, *59*, 1758–1775. <https://doi.org/10.1103/PhysRevB.59.1758>.

[16]	S. Grimme, J. Antony, S. Ehrlich, H. Krieg, “A consistent and accurate ab initio parametrization of density functional dispersion correction (DFT-D) for the 94 elements H-Pu” *J. Chem. Phy.* **2010**, *132*, 154104. <https://doi.org/10.1063/1.3382344>.

[17]	H. J. Monkhorst, J. D. Pack, “Special points for Brillouin-zone integrations” *Phys. Rev. B* **1976**, *13*, 5188–5192. <https://doi.org/10.1103/PhysRevB.13.5188>.

[18]	G. Mills, H. Jónsson, G. K. Schenter, “Reversible work transition state theory: application to dissociative adsorption of hydrogen” *Surf. Sci.* **1995**, *324*, 305–337. <https://doi.org/10.1016/0039-6028(94)00731-4>.

[19]	H. Jónsson, G. Mills, K. W. Jacobsen in *Classical and Quantum Dynamics in Condensed Phase Simulations*, World Scientific, **1998**, pp. 385–404. <https://doi.org/10.1142/9789812839664_0016>.

[20]	G. Henkelman, B. P. Uberuaga, H. Jónsson, “A climbing image nudged elastic band method for finding saddle points and minimum energy paths” *J. Chem. Phy.* **2000**, *113*, 9901–9904. <https://doi.org/10.1063/1.1329672>.

[21]	R. Iftimie, P. Minary, M. E. Tuckerman, “Ab initio molecular dynamics: Concepts, recent developments, and future trends” *Proc. Nat. Aca. Sc.* **2005**, *102*, 6654–6659. <https://doi.org/10.1073/pnas.0500193102>.

[22]	S. Nosé, “A unified formulation of the constant temperature molecular dynamics methods” *J. Chem. Phy.* **1984**, *81*, 511–519. <https://doi.org/10.1063/1.447334>.

[23]	T. Parker, Y. Zhang, K. Shevchuk, T. Zhang, V. Khokhar, Y.-H. Kim, G. Kadagishvili, D. Bugallo, M. Tanwar, B. Davis, et al., “In Situ Raman and Fourier Transform Infrared Spectroscopy Studies of MXene−Electrolyte Interfaces” *ACS Nano* **2025**, *19*, 22228–22239. <https://doi.org/10.1021/acsnano.5c03810>.

[24]	M. Dion, H. Rydberg, E. Schröder, D. C. Langreth, B. I. Lundqvist, “Van der Waals Density Functional for General Geometries” *Phys. Rev. Lett.* **2004**, *92*, 246401. <https://doi.org/10.1103/PhysRevLett.92.246401>.

[25]	J. Klimeš, D. R. Bowler, A. Michaelides, “Van der Waals density functionals applied to solids” *Phys. Rev. B* **2011**, *83*, 195131. <https://doi.org/10.1103/PhysRevB.83.195131>.
